# Religion and Breastfeeding Reconsidered: Exploring the Roles of Congregational and Community Support Along the Mississippi Gulf Coast

**DOI:** 10.3390/healthcare14152380

**Published:** 2026-08-04

**Authors:** John P. Bartkowski, Katherine Klee, Xiaohe Xu, Jacinda B. Roach, Shakeizia (Kezi) Jones

**Affiliations:** 1 Department of Sociology and Demography, University of Texas at San Antonio, San Antonio, TX 78249, USA; xiaohe.xu@utsa.edu; 2 Bartkowski & Associates Research Team, San Antonio, TX 78258, USA; katie@bartconsulting.org; 3 Department of Nutrition and Hospitality Management, School of Applied Sciences, The University of Mississippi, Oxford, MS 38677-1848, USA; jbroach@olemiss.edu; 4 Myrlie Evers-Williams Institute, School of Population Health, The University of Mississippi Medical Center, Jackson, MS 39216, USA; 5 Mississippi Public Health Institute, Madison, MS 39110, USA; kjones@msphi.org

**Keywords:** human lactation, religiosity, spirituality, maternal health, infant health, faith community, social acceptance, lactation professionals, pastoral, church

## Abstract

**Highlights:**

**What are the main findings?**

- Congregational support for breastfeeding is directly and positively associated with the prevalence of breastfeeding in social networks.
- Congregational support for breastfeeding is related to a broader community endorsement of breastfeeding, which in turn is significantly associated with breastfeeding social network prevalence.

**What are the implications of the main findings?**

- The positive association of religious support for breastfeeding with both breastfeeding social network prevalence and broader community support for breastfeeding offers insight into the significant role of religious communities in fostering maternal and infant health. These results could be conceptualized as a collaborative support process to aid in future research explorations across social institutions, namely, family, religion, and infant healthcare: perceived congregational support is linked to increased community support and breastfeeding social network prevalence, while higher levels of breastfeeding social network prevalence may, in turn, expand knowledge-sharing and more favorable discourse within both congregational and community contexts.
- Expanding community-based breastfeeding promotion efforts to include religious congregations may further enhance breastfeeding social network prevalence, particularly in regions with religious and racial demographic compositions similar to those of the Mississippi Gulf Coast.

**Abstract:**

**Background/Objectives**: Previous research has linked religious involvement to increased rates of breastfeeding initiation and duration, as well as improved lactation-related health outcomes. However, affiliation with certain faith traditions may also constrain breastfeeding practices due to misconceptions, modesty norms, religiously informed cultural beliefs, or other contextual factors. Given Mississippi’s status as one of the most religious states in the United States, this study examines unique facets of the association between religion and breastfeeding, with special attention to the direct and indirect role of congregational support. **Methods**: This study uses data from men and women, primarily White and African American respondents, in Wave II of the Mississippi REACH Social Climate Survey. This survey features both established and novel measures of breastfeeding outcomes, enabling new insights into the linkages among religion, community perceptions, and breastfeeding prevalence in primary social networks. The REACH Social Climate Survey combines a general population sample with an oversample of African American residents from the Mississippi Gulf Coast, collected in 2023. The analysis focuses on the direct and indirect associations between congregational support for breastfeeding and the prevalence of breastfeeding within social networks. **Results**: The main findings reveal (1) a direct and positive association between congregational support and breastfeeding social network prevalence and (2) an indirect association between congregational support and breastfeeding social network prevalence through community support for breastfeeding. **Conclusions**: These results underscore the importance of examining the intersectional associations among religion, community context, and infant health outcomes.

## 1. Introduction

Historically, Mississippi has ranked among the most religious states in the United States while simultaneously experiencing pronounced disparities in maternal and infant health outcomes. Approximately 77% of adults in Mississippi identify with a Christian denomination while less than one percent identify as either Muslim, Buddhist, or Hindu. Consequently, 61% of Mississippians report that religion is “very important” in their lives, and nearly 50% attend religious services at least once per week [1]. In past research, Mississippi’s robust religiosity is closely bound to the high proportion of African Americans or Blacks in the state, with this minority group’s religiosity commonly linked to the presence of social stressors and racism, often overwhelmingly present in Southern states [2,3]. In 2025, the Mississippi State Department of Health declared a public health emergency related to infant mortality, citing a rate of 9.7 deaths per 1000 live births and more than 3500 infant deaths occurring before reaching one year of age between 2014 and 2024 [4]. This mortality rate for White infants (6.9) contrasts starkly with that for African American or Black infants (12.5) and the state overall (8.9) [5]. The infant mortality rate, combined with the rate of inadequate prenatal care (17.9) and preterm birth rate (18.5) for African Americans, demonstrates the distinct need to address healthcare gaps across racial and ethnic groups [5]. Breastfeeding has short- and long-term health benefits for both mother and child. The role of religion in addressing Mississippi’s population health disparities is not to be overlooked.

A growing body of research has documented both beneficial and adverse associations between religious involvement, including denominational affiliation, beliefs, and practices, and maternal and infant health outcomes [6,7]. Infant mortality rates are dependent on a variety of social and health factors, including population proportions of religious group involvement and variations across religious denominations. For example, regions with higher proportions of mainline Protestant adherents tend to exhibit lower infant mortality rates compared to areas with greater concentrations of conservative or fundamentalist Protestants net of various controls [6]. Mississippi currently reports the highest infant mortality rate in the United States (8.9 per 1000 live births, compared with a national rate of 5.6), ranking 52nd among all states and U.S. territories [5].

Breastfeeding practices represent a critical and modifiable factor associated with infant survival. Extensive evidence indicates that breastfeeding initiation and continuation are inversely associated with infant mortality, especially in regions characterized by high baseline infant mortality and low breastfeeding prevalence [8]. In Mississippi, approximately 46% of infants are breastfed through their first six months, and only 24% are exclusively breastfed at six months, figures that fall well below national averages of 62% and 28%, respectively [9]. Beyond infant health, breastfeeding confers substantial long-term benefits for maternal health, including reduced risks of breast and ovarian cancers and other positive physical health outcomes [10]. Breastfeeding is defined as “the process of feeding a mother’s breast milk to her infant, directly from the breast or by expressing the milk from the breast and bottle-feeding” [11]. In its policy statement, the American Academy of Pediatrics (AAP) recommends that individuals without medical contraindications exclusively breastfeed for at least six months. Continued breastfeeding through 12 months and beyond, when feasible, is considered optimal due to its well-documented nutritional and health benefits for both mother and infant. At the global level, the World Health Organization (WHO), in collaboration with UNICEF and other international partners, reaffirmed the importance of breastfeeding through the 2005 Innocenti Declaration, which sought to strengthen infant nutrition policies and promote breastfeeding practices worldwide, particularly in regions that have experienced declining infant and maternal health outcomes [12].

Breastfeeding functions as both an individual and a collective practice. At the individual level, the decision to initiate and continue breastfeeding ultimately resides with the mother. However, breastfeeding is also shaped by collective influences, as social support systems, community-based healthcare education, and cultural traditions play critical roles in facilitating initiation and sustaining duration [13,14]. A broad range of social determinants of health, such as educational attainment, housing stability, food availability and security, family structure, marital status, and employment conditions, further shape breastfeeding exposure and norms within familial and social networks [15,16]. In addition, maternal decision-making is influenced by intersecting factors such as culturally specific infant feeding practices, maternal and infant health status, feminist perspectives, political ideologies, and religious identity [17,18,19]. These dynamics underscore the inherently intersectional nature of breastfeeding. Extensive research has also documented persistent racial disparities in breastfeeding practices, with Black women consistently reporting lower rates of breastfeeding initiation and duration compared to their White counterparts [20,21,22,23,24,25,26].

Given the role of race in health disparities and its impact on religious ecologies at the state and community levels, the intersection of race, religion, and breastfeeding is worth exploring. Recent research has revealed distinct patterns that differ from those observed in studies of race and breastfeeding alone. The current study focuses on the intersections of religious (congregational) support, community support, and the prevalence of breastfeeding in social networks. However, it is notable that more than 40% of the study sample identifies as African American, reflecting the demographic composition of Mississippi (37% identify as African American) [27]. Accordingly, attention to the intersecting relationships of race, religious affiliation, and perceived breastfeeding support remains essential. Among Black Christian adults, nearly 90% report recommending six months of exclusive breastfeeding within their family and social networks, and approximately 95% indicate that they would actively support others’ decisions to exclusively breastfeed [28]. Similarly, Black Muslim and Middle Eastern immigrant Muslim communities demonstrate favorable attitudes toward breastfeeding, with correspondingly higher breastfeeding rates relative to the broader, religiously unaffiliated Black population [29,30]. Islamic religious beliefs are often a strong determinant for choosing to initiate and continue breastfeeding, with duration outlasting that of Christian groups [31]. An issue that may arise is varied interpretations of religious texts, which may jeopardize the mother’s health and ability to breastfeed, such as an extended practice of fasting during lactation [30].

Religion as a sociodemographic factor has been identified as directly and indirectly associated with breastfeeding [32]. Research has further demonstrated both facilitators and barriers for breastfeeding practices and perspectives linked to religious contexts [32,33]. General motivating factors include religious texts, beliefs regarding the spiritual or natural qualities of breastmilk, personal prayer practices, and support from faith communities. Conversely, certain doctrinal misinterpretations or cultural influences, such as concerns about milk sharing, beliefs surrounding colostrum, expectations regarding sexual abstinence or fasting, norms of women’s modesty in the presence of men, and cultural beliefs such as the “evil eye,” may serve as barriers to breastfeeding in specific populations [32]. Frequency of religious service attendance has also been associated with an increased likelihood of breastfeeding initiation; however, this relationship appears to be less pronounced with respect to breastfeeding duration [33].

Theological foundations as motivating factors for breastfeeding across religious groups are also worth exploring and provide an important context for this study’s investigation. Augustinian interpretations of scripture propose that Catholic doctrine on kenosis, or the emptying of Christ to become human, can promote breastfeeding as an aspect of self-giving love [34]. Additionally, while the Catholic Church does view formula feeding as a viable option, breast milk, whether shared directly, through stored means, or donated from a milk bank, is the most recommended. Catholic authors promoting breastfeeding often cite encyclicals and health experts on the physical health benefits for mother and infant, and the spiritual sanctuary denoted through family bonding as the first pro-life activity after birth [35]. Marian art, or images and sculptures of Mary, the mother of Christ, depicting Mary breastfeeding, is also prevalent and revered among Catholics as representing an ideal maternal–infant relationship [36]. In Islamic traditions, breastfeeding is closely related to religiously significant beliefs and values [37]. Muslim mothers often view breastfeeding as a divine attribute with spiritual rewards and forgiveness of sins experienced during the process [38]. The practice of breastfeeding is also regarded as sacred in Islam, whereby moral traits are transferred from mother to infant through breast milk [37,38]. In Hindu Ayurvedic texts, or the traditional holistic medical system, breastfeeding is precariously balanced between strict regulations and divine reverence [39]. While the system texts acknowledge breastfeeding as a sacred duty of mothers, there is also a scriptural caution against breastfeeding for mothers who are deemed “unfit” to do so due to particular health conditions and circumstances (i.e., hungry, grieving, emaciated, obese, fever-ridden, pregnant).

While involvement in many faith traditions is associated with a higher likelihood of breastfeeding initiation and, in some cases, longer duration, there exists some variation across more conservative Christian denominations [40]. For example, Catholic women, often categorized with more traditional or conservative faith groups, are more likely to exclusively formula feed compared to women from other denominational groups, with some Protestant women exhibiting similar patterns [41,42]. These results contradict much of Catholic teaching [34,35,36]. Additionally, spiritual but unaffiliated individuals may exhibit distinct infant feeding behaviors, particularly in decisions on breastfeeding initiation [43]. Some religious denominations adhere to stricter health guidelines and may encourage consultations with religious congregational leadership, especially related to breastfeeding initiation and duration. Among Orthodox Jewish groups, tailoring breastfeeding care to guide pumping schedules, use of breast pumps, and religious leader advisement on milk saving are key areas to be addressed by families and healthcare providers [44].

Additionally, the variation in breastfeeding guidance offered to mothers can act as either a support or barrier influenced by religious interpretations, breastfeeding-related misbeliefs, or dismissiveness of breastfeeding concerns or problems [30]. In these contexts, the intersection of community-based education with supportive religious teachings is a critical factor related to breastfeeding attitudes. Cultural competency in healthcare practitioners extends to religious sensitivity [38]. Blending healthcare education with knowledge of religious norms could increase successful health activities in patients. Collaborations between healthcare providers and religious leaders could successfully bridge the gap between religion as a social institution and the communities within which it resides. High levels of perceived support within religious congregations have been attributed to religious leadership endorsement, doctrinal teachings, and shared knowledge emphasizing the natural and health-promoting attributes of breastfeeding [30,31,32,33,34,35,36,37,38,39]. As with other health behaviors, maternal religious practices and affiliations are linked in complex ways to breastfeeding perceptions and experiences.

The current study extends existing research on the association between religion and breastfeeding through an intersectional framework that integrates perceived religious community support, broader community support, and breastfeeding social network prevalence among coastal Mississippians. Drawing on the prior literature linking religious involvement to maternal health behaviors, the primary hypothesis (1) posits that perceived congregational support will be positively associated with the prevalence of breastfeeding within adult Mississippians’ social networks. This hypothesis is related to many religious denominations offering significant social experience opportunities, emotional support, and breastfeeding education, accepting this practice as optimal for child growth [28,29,30,31,32,33,34,35,36,37,38,39,40,43,44]. An ancillary hypothesis (2) suggests that social network prevalence will be positively associated with community support, such that the number of known individuals who have breastfed in a network will be related to the perceived support for breastfeeding from the community. This ancillary hypothesis is formed from the intersecting influences of social norms (agreed-upon rules, ideas, and actionable exchanges), social connections, and the broader community. Overall, the study is driven by the combined hypothesis that perceived religious support will be associated with actual social network prevalence, which will, in turn, be correlated with perceived community support.

These hypotheses are supported by the theory of intersectionality as applied to religion and public health contexts [45,46]. Research on health inequities has traditionally emphasized dimensions such as race, class, and gender. However, this study’s focus on religious involvement represents a critical, yet often underexamined, axis of social differentiation that may structure and determine health behaviors and outcomes. To the authors’ knowledge, perceived religious group support has not been previously examined in conjunction with both breastfeeding social network prevalence and broader community support for lactation. Moreover, religious involvement is a critical dimension of intersectionality in health research, though it is sometimes overlooked in favor of more commonly studied forms of social differences. However, the interconnected roles of community context in breastfeeding social network prevalence, religious influence on perceived support for breastfeeding, and the embeddedness of religion as a major social institution that is influential within community life suggest that these domains are mutually constitutive. As such, the intersection of perceived religious support, community support, and social network prevalence provides a theoretically and empirically valuable framework. In addition, the exploratory nature of this study is a sound basis for examining the social and institutional perceptions of breastfeeding and, more broadly, maternal and infant health support.

## 2. Materials and Methods

The current study was conducted as part of the first phase of the Mississippi Racial and Ethnic Approaches to Community Health (REACH) project, which was implemented for five years, between 2018 and 2023. This analysis draws on validated data from the second wave of a repeated cross-sectional survey, the Mississippi REACH Social Climate Survey Wave II. This survey was fielded for four months during the latter stage of REACH project implementation, between September and December of 2023. The survey was designed to capture perspectives on nutrition, religion, and community support as they related to breastfeeding and infant formula use, alongside key sociodemographic characteristics, including household income, educational attainment, and racial identity, among other factors. Although the cross-sectional design precludes causal inference, it enables the examination of associations between dependent and independent variables while controlling for confounding factors.

The second wave of the survey included refinements and expansions of measures related to congregational breastfeeding support, personal breastfeeding support, and infant feeding knowledge; accordingly, Wave I data were excluded from the present analysis. The CDC-funded Mississippi REACH project prioritized health promotion and the reduction in health disparities disproportionately affecting African American populations in three counties along the Mississippi Gulf Coast. Consistent with this focus, the study sample was drawn from the three Mississippi counties (Hancock, Harrison, and Jackson) served by the REACH project. The overarching goal of the Social Climate Survey was to enhance understanding of racial disparities and social inequalities, particularly between White and African American populations, in breastfeeding and related health outcomes within the coastal region. While we are careful not to generalize beyond the REACH catchment region, findings from this tri-county context may also offer insights applicable to other regions with a similar demographic profile and health disparities.

The study team’s general population survey data collection operated under approval from the Institutional Review Board of Mississippi State University (IRB-17-04-MOU). The analytical sample used in this study was derived from a subsample of the Wave II dataset. While the broader dataset has been utilized in prior research examining the 2022 infant formula shortage and perceived breastfeeding support [26], the present study is distinct in its specific focus on the associations between religious support and breastfeeding social network prevalence in southern Mississippi.

### 2.1. Data

This study utilizes data from the second wave of the Mississippi REACH Social Climate Survey, which combines a general population sample administered in 2023 by the Survey Research Laboratory at Mississippi State University in collaboration with the Mississippi Public Health Institute, along with a non-random oversample of African American adult residents in the study locale. The survey collected detailed information on respondents’ behaviors and attitudes related to nutrition, tobacco use, and infant feeding practices. Key measures relevant to the present analysis include indicators of breastfeeding social network prevalence, perceived (religious) congregational and community support for breastfeeding, and a range of sociodemographic characteristics.

### 2.2. Sampling and Data Collection

**Figure 1:**
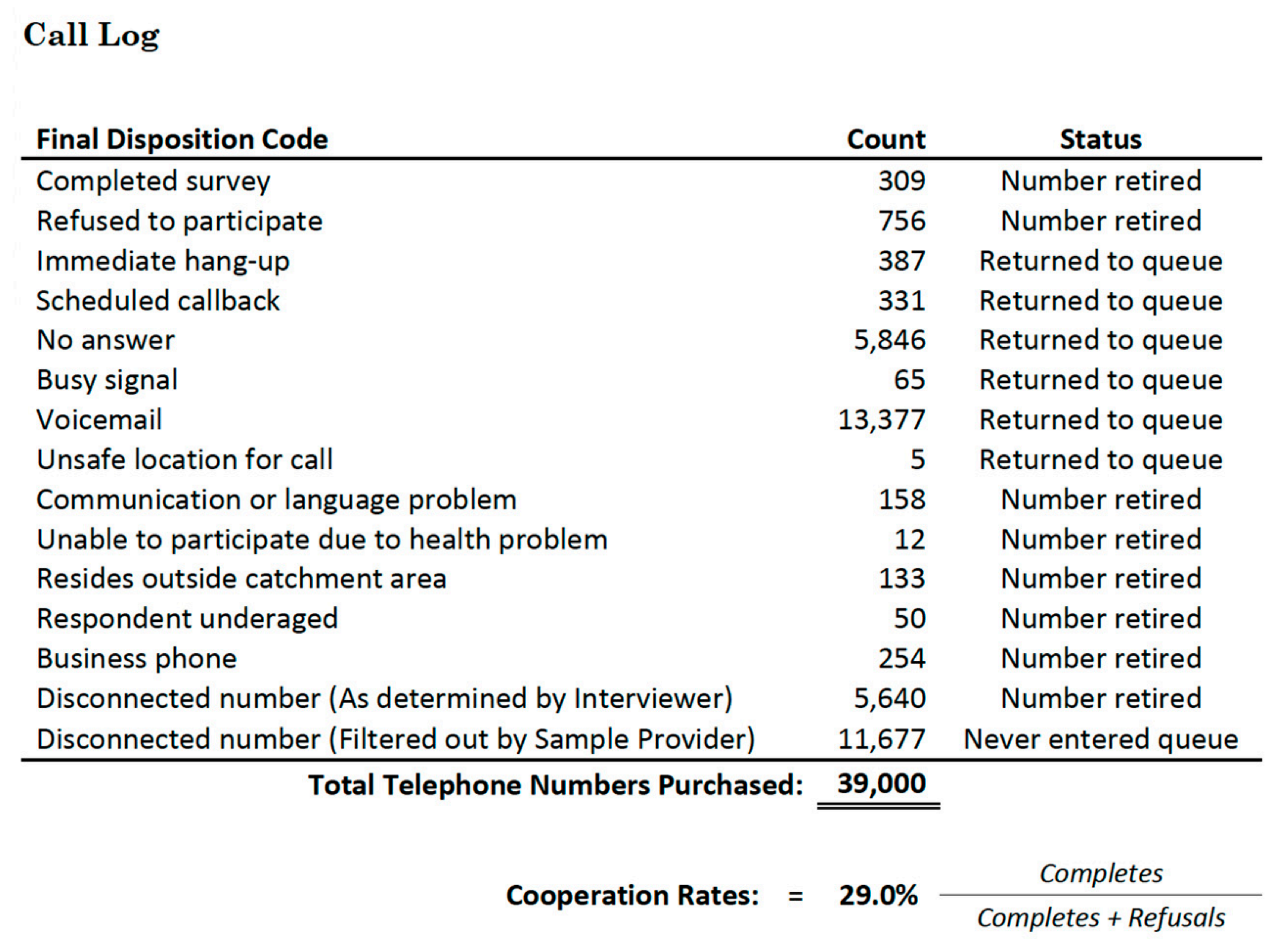
Call log generated by the Survey Research Laboratory at Mississippi State University in collaboration with the Mississippi Public Health Institute; displays the tracked phone survey administration.

The general population sample participated in a telephone survey administered to a representative sample of adults aged 18 and older residing in the Gulf Coast region, including Jackson, Hancock, and Harrison counties. Providing a probability-based sample representative of all households in the three-county catchment area required the use of a dual-frame RDD (Random-Digit-Dialing) sampling methodology, whereby both landline and cellular telephone numbers were used to contact eligible adults. System missing codes in the SPSS (version 30) dataset and Item Response Frequency Tables indicate that a question was not asked of a given respondent because it did not apply based on his or her pattern of responses. For dichotomous response options with a 50/50 split, the margin of error for the total dataset (*n* = 309) is no larger than ±6% (95% CI). As seen in Figure 1, telephone numbers were dialed a maximum of eight (8) times before being retired, unless a final disposition was secured prior to the eighth call. While county-level and survey participant landline versus cellular use information is unavailable, 2018 state-level data indicate 66.5% of households register as cellular use only, 14% as mostly cellular use, 4% as mostly landline use, and 3.4% as no telephone service use [47]. Therefore, the use of both landline and cellular phone numbers, along with online surveying for the African American oversample, covered the broadest range of population sampling possible for the designated region. This general population sample was supported by an African American oversample of adult males and females 18 years of age and older.

To oversample African American respondents, an additional non-probability sample of 118 African American adults was selected by the Mississippi Public Health Institute using a purposive sampling technique through SurveyMonkey, an online survey platform, and ensured complete parity across both general population and oversample population surveys. The oversample survey collection was administered through MS REACH partner organizations (i.e., congregations, community centers, senior centers), with outreach completed by an in-person team member across the three Gulf Coast counties. Rendering a precise completion rate for the oversample was not prioritized to avoid over-burdening community partners with data collection tracking efforts. However, it is estimated that there was a relatively smaller proportion of survey refusals than the general population survey due to the collaboration with trusted African American health advocates in the communities. Incentives of USD 10 Amazon e-gift cards were offered per completed oversample survey, with the addition of a random drawing raffle for one of three USD 100 Amazon e-gift cards, to all qualifying oversample participants.

It is worth noting that the survey was linked for a short time to a website by a partner organization and resulted in 1301 bot completions. Each bot completion was identified and removed prior to data analyses. Safeguards were immediately pursued following the notice of bot completions to ensure that only Mississippi Gulf Coast residents completed surveys and obtained the incentives. A short follow-up survey was administered to the African American oversample participants to capture a more in-depth perspective on the relationships between the selected independent and dependent variables and create full parity with the general population survey. The current study uses de-identified data from the random general population sample and the purposive oversample combined. Participant inclusion criteria were taken from the African American and White racial categories, age 18 years or older, gender (male, female, or other), and Mississippi REACH three coastal counties as the location. Exclusion criteria were younger than age 18 and/or not a resident of the three counties within the study’s parameters. All excluded individuals were notified of their ineligibility to complete the survey and discontinued from participation. All qualifying participants were clearly informed that they could skip any item, item response, or choose to withdraw from survey participation at any point before submission. Participants were also informed of the voluntary nature of the survey and the potential publication of the de-identified responses. In total, Wave II of the Mississippi REACH Social Climate Survey comprises 427 completed telephone and online surveys. For the present analysis, 13 respondents were excluded due to the absence of female friends or relatives with children, resulting in a final analytical sample of 414 adults from the Mississippi Gulf Coast region.

### 2.3. Variables

The dependent variable in this study is the prevalence of breastfeeding within respondents’ social networks, measured through a single survey item: “How common is breastfeeding infants among your female friends and relatives?” Response options included: 1 = None of them have breastfed, 2 = Very few of them have breastfed, 3 = About one quarter of them have breastfed, 4 = About half of them have breastfed, 5 = A majority of them have breastfed, and 6 = All of them have breastfed, 7 = None of my female friends or relatives have any children, 8 = I don’t have any female friends or relatives, 9 = Don’t know, and 10 = Refused to answer. As noted previously, responses in categories 7 and 8 were excluded from the analytical sample, while responses of “Don’t know” and “Refused” were recoded as missing. The term “breastfeeding social network prevalence” is used to define the amount of exposure to breastfeeding that our study participants have experienced through family members, friends, coworkers, and other social connections who have breastfed. This term and corresponding variable have been included in previous publications using the Social Climate Survey from Mississippi REACH. This variable intentionally does not gauge respondents’ personal breastfeeding practices. Doing so would only be applicable to women and thereby compromise our statistical power through massive case attrition. Therefore, a prevalence measure gauges the pervasiveness of breastfeeding in respondents’ primary social networks while preserving our sample size.

The key independent variable is perceived congregational support for breastfeeding, assessed by a single item on a 5-point Likert scale: “In your opinion, how supportive of breastfeeding are people in your church, congregation, or faith community?” Responses are 1 = Very unsupportive, 2 = Somewhat unsupportive, 3 = Neither unsupportive nor supportive, 4 = Somewhat supportive, and 5 = Very supportive. The mediating variable, perceived community support for breastfeeding, was measured using a parallel 5-point Likert scale. Respondents were asked: “In general, how supportive of breastfeeding are people in your community?” with response options: 1 = Very unsupportive, 2 = Somewhat unsupportive, 3 = Neither unsupportive nor supportive, 4 = Somewhat supportive, and 5 = Very supportive. Several sociodemographic characteristics were included as control variables, with dummy-coded male, other marital statuses, white, not working, and adults aged 65 and older serving as reference categories. Household income in 2022 (ranging from 1 = Less than $10,000 to 11 = $200,000 or more) and educational attainment (ranging from 1 = Never attended school or only attended kindergarten to 9 = Beyond master’s degree) were treated as continuous measures in the analysis.

### 2.4. Analytic Strategies

To evaluate the conceptual model examined in this study, missing values in both the dependent and independent variables were imputed under the assumption of missing at random (MAR) (see Table 1). A multiple imputation method was employed to estimate missing values and avoid selection bias. The procedure used was the chained equations method through linear regression, because the dependent variable was treated as continuous. To safeguard the path analysis results, a listwise deletion of all missing values was also implemented, followed by an additional set of path analyses. Consistent results were achieved utilizing these methods. While all path coefficients remained statistically significant, the levels of significance decreased as sample size and statistical power declined. Nevertheless, these additional checks and auxiliary analyses demonstrated that the results are sound, robust, and replicable.

Before conducting multivariate analyses, descriptive statistics and bivariate Pearson correlation coefficients were computed and reported. A path analysis was subsequently conducted to estimate the direct, indirect, and total effects (variable associations given our use of cross-sectional data) of perceived congregational support for breastfeeding on the prevalence of breastfeeding within social networks among adults residing in the Mississippi Gulf Coast region. The goodness-of-fit indices indicate a perfect fit of the path model (RMSEA = 0; SRMR = 0; CFI = 1; Likelihood ratio χ^2^ = 0). Consistent with routine practice in path modeling, standardized path coefficients were reported to facilitate the interpretation and comparison of effect sizes across variables. All analyses were performed using the Structural Equation Modeling (SEM) module in Stata version 17 [48]. In this paragraph and the sections that follow, the term “effect” is used in accordance with the terminology and logic of path analysis. Although this terminology implies directionality, the cross-sectional design of the study precludes definitive conclusions regarding causal or temporal ordering among variables. Any language of directional influence on our part is presumptive and subject to future research with more robust longitudinal data. This limitation is addressed in greater detail in Section 4.

## 3. Results

Table 2 presents descriptive statistics for all variables included in the study. Among the 414 respondents in the analytical sample, the majority were women (60.63%, *n* = 251). Nearly half of the participants (47.34%, *n* = 196) reported being married at the time of the survey, and a substantial proportion (74.4%, *n* = 308) were between the ages of 18 and 64. Due to intentional oversampling of African American adults, the sample included 41.79% African American respondents (*n* = 173), exceeding the 37.9% representation of African Americans in Mississippi reported in the 2020 Census [28]. On average, respondents reported having completed some college education or vocational training. The average household income fell between the “$35,000 to < $50,000” and “$50,000 to < $75,000” categories.

Turning to the dependent variable, respondents reported that, on average, between one-quarter and one-half of the women in their social networks had breastfed, indicating a moderate level of breastfeeding social network prevalence. With respect to perceived support for breastfeeding, both congregational and broader community support for breastfeeding were, on average, situated below the “somewhat supportive” category but above “neither supportive nor unsupportive.” This pattern suggests a generally favorable, albeit not strongly affirmative, social climate toward breastfeeding.

Table 3 presents the bivariate Pearson correlation coefficients for all variables included in the path analysis. As expected, congregational support, community support, and breastfeeding network prevalence are positively correlated. Although the magnitude of these coefficients is relatively small (≤0.35), they are statistically significant at the 0.001 level. Furthermore, being married, being African American, household income, and educational attainment are significantly correlated with congregational support, community support, and breastfeeding network prevalence (at least at the 0.05 level). The only exception is the correlation between educational attainment and breastfeeding prevalence within social networks, which is not statistically significant. Collectively, these correlations provide a strong empirical basis for the proposed path analysis while accounting for sociodemographic characteristics.

**Figure 2:**
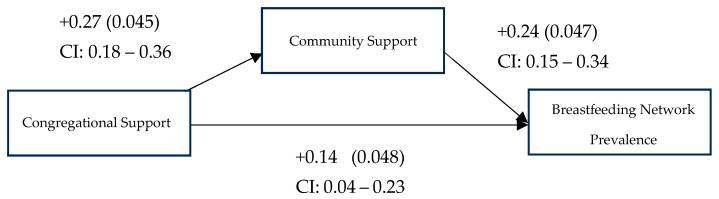
Effects of Congregational Support on Breastfeeding Network Prevalence.

Table 4 presents the standardized path coefficients corresponding to the conceptual model depicted in Figure 2. Three key findings emerge from the analysis. First, consistent with the primary study hypothesis, congregational support for breastfeeding is directly and positively associated with the prevalence of breastfeeding within social networks. The standardized path coefficient of 0.14 indicates that a one standard deviation increase in perceived congregational support is associated with a 0.14 standard deviation increase in breastfeeding network prevalence, controlling for sociodemographic characteristics. This association is statistically significant at the *p* < 0.001 level.

Second, the analysis reveals an indirect positive effect (association) of congregational support for breastfeeding on the prevalence of breastfeeding networks, mediated by perceived community support. The standardized path coefficient from congregational support to community support is 0.27 (*p* < 0.001), and the indirect effect (association) from perceived community support to breastfeeding prevalence is 0.06 (*p* < 0.001), indicating that congregational support contributes to a broader community endorsement of breastfeeding, which in turn is associated with greater prevalence of breastfeeding within social networks.

Finally, the total effect (association) of congregational support for breastfeeding, encompassing both direct and indirect pathways, is positive (0.20) and statistically significant (*p* < 0.001). Taken together, these findings lend empirical support to the proposition that religious norms surrounding breastfeeding exert both direct and indirect influences on breastfeeding social network prevalence, primarily through their impacts on broader community attitudes and norms. As such, the path model illustrated in Figure 2 is substantiated by survey data collected from the Mississippi Gulf Coast region. Once again, we acknowledge that directional relationships among key variables are presumptive based on path analysis with cross-sectional data and require additional examination in future research with time series data.

## 4. Discussion

Mississippi continues to rank among the most religious states in the United States while simultaneously experiencing some of the poorest health outcomes, including the highest rates of infant mortality and lowest rates of breastfeeding [1,2,3,4,5]. The state’s large population of religiously affiliated Black adults, comprising approximately 40% of both the general population and the study sample, faces a disproportionate burden of health disparities and lower rates of breastfeeding initiation compared to non-Black Mississippians [5,9,28]. In response, recent community efforts have emerged to promote breastfeeding education among populations at elevated risk for adverse maternal and infant health outcomes [23]. The implications of these actions are reflected in community-level attitudes captured in datasets such as the Mississippi REACH Social Climate Survey [25,26]. The findings from the current study indicate that perceived support for breastfeeding within both congregations and the broader community is generally favorable. An association is also clear between perceived congregational breastfeeding support and breastfeeding social network prevalence among coastal Mississippians. These results are consistent with the primary hypothesis that predicted congregational support for breastfeeding would be positively associated with greater social network prevalence of breastfeeding. However, our ancillary hypothesis was not as strongly supported. We hypothesized that social network prevalence would be positively correlated with broader community support, mediating on behalf of the religious community; instead, congregational support is directly and positively associated with both community support and breastfeeding social network prevalence. Congregational support is also indirectly associated with social network prevalence through community support, which is positively associated with prevalence in social networks.

The findings suggest a central role of perceived religious support for breastfeeding in relation to (1) actual social network prevalence (breastfeeding connections) and (2) perceived community support for breastfeeding. Perceptions of community support, while still linked to social network prevalence, appear somewhat less pronounced than perceived congregational support. It can be inferred that congregational perspectives on breastfeeding are related to the connections made during breastfeeding and the overall community’s breastfeeding-friendliness, supported by previous research [29,30]. Congregational breastfeeding supports would seem to be associated with positive community perceptions of lactation along with increased social network prevalence. We anticipate, but are unable to empirically test, that such factors would be most robust among frequently attending congregants or those with strong social ties within the congregation due to more intensive messaging exposure. Robust support for breastfeeding across communities remains a central goal for healthcare providers and lactation professionals. In Mississippi, the implementation of three out of the six recommended initiatives, such as Medicaid coverage through one year postpartum, mandatory postpartum depression screenings, and maternal mortality review committees, suggests that a foundation of moderate systemic support is in place [5]. The findings of the current study suggest that faith-based support for breastfeeding, most likely across Christian Protestant denominations as nearly three-fourths (71%) of Mississippians identify as such [1], may be an influential factor in perceived community support. Receiving emotional support and breastfeeding education from fellow congregation members is a significant element in African American Christian favorability towards breastfeeding [28]. However, biblical evidence of breastfeeding as the ideal source of nourishment for infants and pastor-driven support are also impactful factors. Breastfeeding role modeling in scripture and supportive language from pastors at the pulpit are critical elements for shaping lactation perspectives in majority-African American Christian congregations [28]. Perceived community support, in turn, exhibits a significant association with social network prevalence. The survey instrument used in this study did not capture denominational differences or qualitative data on individual experiences with perceived breastfeeding support. Therefore, it is important to be aware of variations in religious orientations toward the body and, by extension, breastfeeding. Much of the pro-breastfeeding discourse found in denominations described in the literature review is either absent or contradicted in many conservative Protestant (fundamentalist) congregations. This pattern is likely due to their theological views of the body as sinful and depraved, along with norms that stress modesty in all forms of bodily comportment [40]. Therefore, a spectrum of theologically informed orientations toward breastfeeding is exhibited across denominational contexts. Still, where support for breastfeeding is evident, several plausible components of moderate support can be identified. Congregational supports may include the availability of comfortable and secure lactation spaces in places of worship; the use of communal spaces for breastfeeding support groups, educational sessions, or breastfeeding-friendly family-centered events; and the reduction in stigmatizing language or myth-based information-sharing. Removing stigmatizing language in congregations, places of perceived acceptance, guidance, and social influence could be connected with greater support in the broader community.

The dissemination of accessible resources and accurate information on breastfeeding, particularly within congregational settings, may contribute to more favorable attitudes towards breastfeeding in communities disproportionately affected by health disparities [2,13,14,40]. Therefore, the role of congregations across denominations should be considered a significant factor in breastfeeding promotion and education strategies. Alternatively, family members, friends, co-workers, and others may not attend the same congregations or reside in the same communities. Congregational support alone should not be assumed as the principal correlate of community support for breastfeeding or the commonness of breastfeeding in primary social networks. More so, the broad array of intersecting social networks allows for greater collaboration and idea dissemination. In other words, support from one congregation can be viewed and shared by individual members with members from other congregations, all of whom may share these perspectives across their social networks that span workplaces, community centers, and more. This perspective lends credence to the results that perceived congregational support for breastfeeding is positively associated with both perceived community support and social network prevalence. Ultimately, fostering widespread awareness, promoting factual knowledge-sharing, and strengthening peer support opportunities are likely to improve perceptions of breastfeeding. This moderate level of congregational and community engagement signifies a meaningful pathway through which public health practitioners and lactation advocates can build upon existing progress. Continued and coordinated efforts across both secular and faith-based settings may further enhance breastfeeding outcomes and contribute to improved maternal and infant health.

The results further suggest the possibility of a collaborative, cross-sector support process through an intersectional lens. The observed modest associations among perceived congregational support for breastfeeding, community support for breastfeeding, and breastfeeding social network prevalence are novel to this study, and the potential for collaborative relationships warrants consideration for future research. As depicted in Figure 2, perceived congregational (religious) support exhibits both direct and indirect associations with breastfeeding social network prevalence, and community support serves as a moderating pathway that amplifies the association. Such an intersectional relationship implies that as breastfeeding is supported through visible and social means in small congregations and larger communities, it may foster increasingly supportive perceptions in both active and passive members. Simply observing social acceptance can be a marked shift in behavioral attitudes. This process may be facilitated through knowledge-sharing, the correction of persistent misconceptions, and the development of peer support networks both within and beyond established social groups. These social interactions can create opportunities for dialogue, experiential learning, and the diffusion of positive norms surrounding breastfeeding. From a policy perspective, the current findings highlight the intersectional roles of religion, community context, and maternal decision-making in shaping infant feeding practices. Breastfeeding represents both an individual choice and a socially embedded behavior. Mothers rely on multiple layers of support, including guidance from healthcare providers, shared experiences with other women, and the broader foundation of social determinants of health (i.e., income, education, access to healthcare, food security, and caregiving resources). Strengthening these interconnected supports, particularly within both secular and faith-based settings, may enhance breastfeeding initiation and duration while also contributing to improved maternal and infant health outcomes [13,14,15,16].

### Limitations and Future Research

The present study contributes to the growing body of literature examining the associations among religious support for breastfeeding, community support for breastfeeding, and the prevalence of breastfeeding within social networks. Nevertheless, several limitations must be acknowledged. First, the use of time series data would be quite promising for future research projects. Implementing a multi-wave model with longitudinal data using the same items for congregational support, community support, and breastfeeding social network prevalence would allow for the study over time to ascertain causal patterns and temporal variations. Because a cross-sectional survey was used in this study, the research team does not assume true causal pathways from congregational support to breastfeeding network prevalence, either directly or indirectly, as the mediation pathway [49]. In addition, the use of self-reported measures incurs the possible influences of memory bias and social desirability bias. However, the use of untimed, online surveys for the African American oversample somewhat mediated for a portion of the study’s sample population compared to other methods, such as interviews or focus groups. Second, future research would benefit from incorporating qualitative methodologies to complement quantitative findings. Approaches such as focus groups, in-depth interviews, and panel discussions could provide more nuanced insights into the lived experiences of perceived breastfeeding support across diverse populations, including variations by religion, gender, race and ethnicity, educational attainment, and socioeconomic status. Such insights would enhance the development of more precisely targeted public health interventions and inform policy at both state and national levels. A focus on methodological approaches for a future study could expand on the differing relationships of response patterns and demographics of time-specific respondents (i.e., those who completed the survey on first contact versus those who completed the survey on a follow-up contact) utilizing a linear analysis by one-way analysis of variance or Pearson correlations.

Third, this study was unable to address denominational variations or comparative findings across major religions (e.g., Hinduism, Buddhism, Islam, Judaism, Christianity) in breastfeeding patterns, which represents an important direction for future inquiry. Introducing historical and theological foci on the role of religion in breastfeeding perspectives would not only benefit the scientific field, but also future cultural competency trainings and care practices. The majority of Mississippians (71%) identify as Christian Protestants. However, exploring county-level variations would be beneficial for exploratory purposes. This study surveyed three counties that were served by the overarching CDC-funded REACH project. Expanding the location population sample would also potentially increase variability in religious affiliation, cultural perspectives, and breastfeeding experiences. Replicating this study in other geographical locations would also improve external validity given the generalizability limits of a three-county exploratory study. Examining potential interactions among locale, gender, race, and denominational affiliation would contribute substantially to the study of intersectionality. Expanding research to incorporate denominational variations by racial/ethnic background and more robust intersectional analyses would strengthen understanding of how religious contexts shape breastfeeding in social networks. Fourth, data collection limitations should also be acknowledged. The initial online distribution of the oversample survey resulted in automated (bot) responses, which were subsequently identified and removed before analysis. To enhance data quality, future research should prioritize more controlled data collection strategies, such as administering surveys in person, through targeted email recruitment, mailed-in documents, or via telephone interviews [50]. Further, while landline use is becoming less prevalent, collecting information on response rates related to landline versus cellular use could refine survey approaches [50]. Finally, while the survey instrument for Wave II underwent content validation informed by input from community partners and residents, future studies would benefit from additional validation efforts tailored to specific subpopulations to further strengthen measurement reliability and cultural relevance.

## 5. Conclusions

Mississippi has historically ranked among the leading states in both elevated infant mortality and high levels of religiosity, making it a particularly salient context for examining social factors central to maternal and infant health. Previous research has established associations between religious involvement and willingness to initiate and sustain breastfeeding. The findings of the present study augment existing scholarship by exploring the relationship between congregational support and community support for breastfeeding on the one hand and breastfeeding social network prevalence on the other. Specifically, moderate levels of perceived support within congregations are positively and directly associated with greater perceived support in broader community contexts and higher social network prevalence of breastfeeding among participants. Additionally, perceived community support for breastfeeding is directly connected to the prevalence of breastfeeding in social networks. These findings suggest that more explicit and widespread congregational endorsements of breastfeeding may contribute to increased community support efforts and breastfeeding social network prevalence. Such support may also facilitate greater dissemination of accurate information about breastfeeding, combatting persistent myths, reducing stigmatizing discourse, and expanding social acceptance of breastfeeding in public settings. Moreover, the results point to promising collaborative processes in which the perception of breastfeeding support in congregations, potentially through safe lactation spaces and breastfeeding-friendly announcements or events, relates to community-level perceptions and exposes members to breastfeeding across their social networks. This process should promote more favorable attitudes, strengthen peer support, and foster greater normalization of breastfeeding across both religious and community contexts. Future research should further investigate denomination-specific differences in breastfeeding initiation and duration, varied geographical study replication, as well as the role of religious institutions in shaping community-level education and social acceptance of breastfeeding. From a policy perspective, interventions are likely to be most effective when they address gaps in breastfeeding education and promote informed, supportive discourse among both community members and healthcare providers. Promotional efforts would do well to develop and evaluate faith-based breastfeeding interventions in collaboration with religious leaders and healthcare professionals. Attention should be directed toward populations with high levels of religious attendance experiencing disproportionate health disparities, where integrated, community-engaged approaches (i.e., faith-based initiatives) may yield meaningful improvements in maternal and infant health outcomes.

## Figures and Tables

**Table 1:** Patterns of Missing Values.

Variables	Missing	%
Breastfeeding	40	9.7
Congregational support	121	29.2
Community support	96	23.2
Gender	25	6.0
Marital status	26	6.3
Race	6	1.4
Age	1	0.2
Income	69	16.7
Education	5	1.2
Employment status	4	1.0

**Table 2:** Sample Characteristics (*n* = 414).

	*n*	Percent	Mean	SD	95% CI	
Breastfeeding Network Prevalence	-	-	3.75	1.47	3.61	3.90
Congregational Support for Breastfeeding	-	-	3.75	1.35	3.62	3.88
Community Support for Breastfeeding	-	-	3.66	1.29	3.54	3.77
Male	163	39.37	-	-	0.35	0.44
Female	251	60.63	-	-	0.56	0.65
Married	196	47.34	-	-	0.43	0.52
Other Marital Statuses	218	52.66	-	-	0.48	0.57
African American	173	41.79	-	-	0.37	0.47
White	205	49.51	-	-	0.45	0.55
Other Races	36	8.7	-	-	0.06	0.12
Older Adults (65+)	106	25.6	-	-	0.21	0.30
Other Age Groups (≤64)	308	74.4	-	-	0.70	0.784
Household Income	-	-	6.34	2.67	6.09	6.61
Education	-	-	5.72	1.75	5.55	5.89
Employed	255	61.59	-	-	0.57	0.659
Not Working/Other	159	38.41	-	-	0.336	0.435

**Table 3:** Pearson Correlation Coefficients.

Variables	1	2	3	4	5	6	7	8	9	10	11
1. Breastfeeding Prevalence	1.00										
2. Congregational Support	0.28 ***	1.00									
3. Community Support	0.35 ***	0.34 ***	1.00								
4. Female	−0.04	0.02	−0.07	1.00							
5. Married	0.11 *	0.18 ***	0.17 ***	−0.02	1.00						
6. African American	−0.27 ***	−0.24 ***	−0.25 ***	0.08	−0.18 ***	1.00					
7. Other Races	0.16 ***	0.02	0.05	0.02	−0.09	−0.23 ***	1.00				
8. Age	0.02	−0.01	−0.05	0.11	0.15 **	0.07	−0.12 *	1.00			
9. Household Income	0.24 ***	0.22 ***	0.20 ***	−0.04	0.35 ***	−0.28 ***	−0.02	0.09	1.00		
10 Education	0.05	0.13 **	0.12 **	0.10 *	0.15 ***	−0.07	0.00	−0.07	0.28 ***	1.00	
11. Employed	0.07	0.05	0.01	−0.09	−0.04	0.04	−0.06	−0.45 ***	0.13 **	0.25 ***	1.00

Note: * *p* < 0.05, ** *p* < 0.01, *** *p* < 0.001.

**Table 4:** Standardized Path Coefficients of Congregational Support to Predict Community Support and Breastfeeding Network Prevalence.

Variables	Direct Effect	*p*-Value	Indirect Effect	*p*-Value	Total Effect	*p*-Value
Congregational Support → Breastfeeding Network Prevalence	0.14 (0.048) CI: 0.04–0.23	0.000	0.06 (0.017) CI: 0.03–0.09	0.000	0.20 (0.048) CI: 0.11–0.29	0.000
Congregational Support → Community Support	0.27 (0.045) CI: 0.18–0.36	0.000				
Community Support → Breastfeeding Network Prevalence	0.24 (0.047) CI: 0.15–0.34	0.000				

Note: The standardized path coefficients are adjusted for sociodemographic characteristics. Standard errors are in parentheses. CI: 95% confidence interval.

## Data Availability

Data are not available due to privacy and ethical restrictions. Because these data are proprietary (i.e., held in a private repository), they are not publicly available or otherwise allowed to be circulated. More information about the data can be provided by the first author upon request.
